# Seasonal fluctuations of *Babesia bigemina* and *Rhipicephalus microplus* in Brangus and Nellore cattle reared in the Cerrado biome, Brazil

**DOI:** 10.1186/s13071-022-05513-2

**Published:** 2022-10-28

**Authors:** Kauê Rodriguez Martins, Marcos Valério Garcia, Paulino Bonatte-Junior, Pâmella Oliveira Duarte, Barbara Guimarães Csordas, Leandro de Oliveira Souza Higa, Namor Pinheiro Zimmermann, Jacqueline Cavalcante Barros, Renato Andreotti

**Affiliations:** 1 Programa de Pós-Graduação em Ciências Veterinárias, Fundação Universidade Federal de Mato Grosso do Sul, Campo Grande, MS 79070-900 Brazil; 2 Embrapa Gado de Corte, Vila Popular, Avenida Radio Maia, 830., Campo Grande, MS 79106-550 Brazil; 3 Programa de Pós-Graduação em Ciência Animal, Fundação Universidade Federal de Mato Grosso do Sul, Campo Grande, MS 79070-900 Brazil; 4 Programa de Pós-Graduação em Doenças Infecciosas e Parasitárias, Fundação Universidade Federal de Mato Grosso do Sul, Campo Grande, MS 79070-900 Brazil; 5 Universidade Federal do Paraná (UFPR), Campus Palotina, Palotina, PR 85950-000 Brazil

**Keywords:** Babesiosis, Beef cattle, Bovine, Tick-borne diseases

## Abstract

**Background:**

The tick *Rhipicephalus microplus* (Ixodida: Ixodidae, Canestrini, 1888) causes substantial economic and health losses in cattle production and is the main vector of *Babesia bigemina* (Piroplasmidae: Babesidae, Smith & Kilborne, 1893). *Babesia bigemina* is responsible for a tick-borne disease known as babesiosis that can cause hemolytic anemia, fever and death. In the study reported here, we investigated the relationship between the number of ticks per animal and the number of *B. bigemina *cytochrome* b* gene (*cbisg*) copies in the blood of Brangus and Nellore cattle reared without acaricidal treatment in the Brazilian Cerrado biome over a 1-year period.

**Methods:**

Ticks on 19 animals (9 Brangus and 10 Nellore cattle) were counted every 18 days, and blood was collected every 36 days for 12 months. Serological samples were analyzed with an indirect enzyme-linked immunosorbent assay, and genomic DNA was analyzed by conventional PCR and quantitative PCR. The PCR products were sequenced by the Sanger method.

**Results:**

The Brangus and Nellore breeds showed similar weight development and no clinical signs of babesiosis. Statistically significant differences (*P* < 0.05) between the breeds were observed for the number of ticks and the number of *B. bigemina cbisg* gene copies.

**Conclusions:**

No correlation between the number of ticks and the number of circulating copies of *cbisg* was observed, although Nellore cattle presented with fewer ticks than Brangus cattle and the number of *cbisg* copies was higher for Nellore cattle than for Brangus cattle.

**Supplementary Information:**

The online version contains supplementary material available at 10.1186/s13071-022-05513-2.

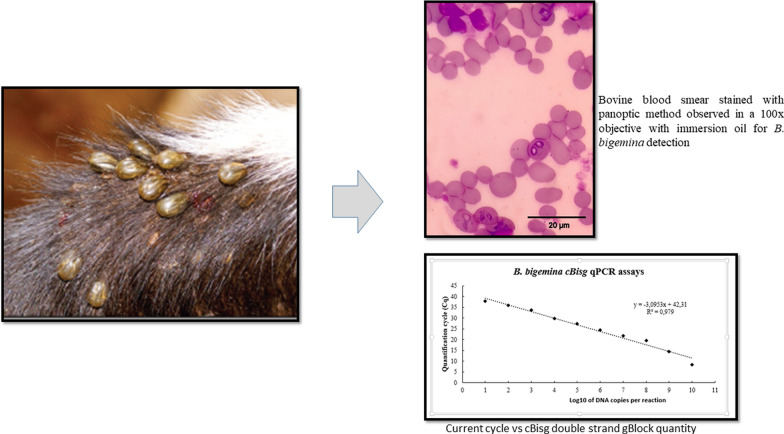

## Background

In the Cerrado biome, the cattle tick *Rhipicephalus microplus* is responsible for the transmission of cattle tick fever (CTF), a complex of diseases caused by three hemoparasites: *Anaplasma marginale *(Order: Ricketisales), a Gram-negative bacterium that causes anaplasmosis in wild and domestic ruminants, and *Babesia bovis* and *Babesia bigemina* (Order: Piroplasmida), protozoans causing babesiosis. These hemoparasites are mainly present in tropical and subtropical regions and may cause clinical signs such as hemolytic anemia, fever, occasional hemoglobinuria and death [1, 2].

A single host can be persistently infected with one more hemoparasites that cause similar clinical signs, making the identification of the cause of CTF difficult in large herds, and leading to a similar, but not completely effective treatment for the disease complex [3]. The Brazilian Cerrado biome provides a favorable environment for cattle breeding [4], but also for *R. microplus* development*.* This tick has a strong preference for cattle, causing large direct and indirect economic losses related to the cattle industry [5].

*Bos indicus* cattle have been found to present fewer engorged ticks than other types of cattle and are known as a breed resistant to tick infestation [6, 7]. They also exhibit lower levels of *Babesia* spp. parasitemia [8] than other taurine breeds and their crosses. However, the degree of parasitism may vary with animal age and breeding system [9]. Crossbreeding with taurine cattle is used to increase the genetic propensity for weight gain and precocity in beef cattle, and Brazil’s Cerrado biome has increased its use of the Brangus crossbreed at the cost of lower resistance to tick infestation [10].

The absence of a well-defined, effective and well-deployed vaccine against babesiosis [11], tick resistance to chemical control [12], the capacity for transovarial *Babesia* spp. transmission in ticks [13] and the endemism of babesiosis in Brazil have all resulted in disease control and monitoring becoming more difficult.

The aim of this study was to investigate the number of ticks and assess *B. bigemina* in naturally infested Brangus and Nellore cattle reared in the growth phase without any acaricide intervention in the Brazilian Cerrado biome.

## Methods

### Study area

The study was conducted in the municipality of Água Clara, Mato Grosso do Sul State, Brazil (20°46′24″S 52°32′24″W; altitude: 309 m a.s.l.). The climate in this area is characterized as humid tropical, with a 1- to 3-month-long dry season and an average temperature of > 18 °C in all months of the year [14]. According to Flumignan et al. [15], the rainfall distribution pattern in the municipality of Água Clara follows a very consistent pattern in which most of the rainfall occurs from December to February, decreasing gradually from March to May until the dry season from June, to August. A gradual increase in rainfall occurs from September to November until the rainiest months of the year. The present study was carried out from June to December 2016, and meteorological data were obtained from the Mato Grosso do Sul state government database [16].

### Animals used in the experiment

Nineteen (9 Brangus and 10 Nellore cattle) pasture-raised animals in the growth phase, aged 8–10 months and with a mean weight of 219.5 kg, were studied. These animals were naturally infested and kept together at a density of 0.6 animals per hectare. The ground consisted of sandy soil with a pasture composed of *Urochloa* (*Brachiaria*) *decubens*. Acaricidal treatment and prophylactic control of tick-borne diseases (TBDs) were not performed.

### Tick count and blood sampling

Data were collected from June 2016 to June 2017, with intervals of 18 days [10] between tick counts according to the method of Wharton and Utech [17]. Briefly, ticks with lengths > 4.5–8 mm on both sides of each animal were counted. Tick taxonomic classification was performed following the system of Pereira et al. [18]. Additionally, every 36 days, the animals were weighed on a digital scale (Coimma®; Dracena—SP, Brazil) to assess animal welfare [19], and blood was harvested from the caudal vein using sterile vacuum tubes containing ethylenediaminetetraacetic acid anticoagulant. In total, 228 blood samples (12 from each animal) were collected; these samples were kept at 4 °C and transported to the laboratory for serum and genomic DNA (gDNA) extraction.

### DNA extraction

Genomic DNA was extracted in duplicate from whole blood as previously described [20]. Briefly, each extraction was performed in a 2-ml micro tube containing 300 μl of bovine blood, 2 μl of proteinase K (20 mg/ml) (Sigma-Aldrich, St. Louis, MO, USA) and 500 μl of sodium dodecyl sulfate (20%). Each sample was incubated for 1 h in a water bath at 65 °C, following which 800 μl of chloroform was added to the micro tube and the samples vigorously vortexed to achieve homogenization. Then, 350 μl of protein precipitation solution (6 ml potassium acetate, 1.1 ml glacial acetic acid, 2.9 ml ddH_2_O) was added, and the mixture was centrifuged at 13,000 rpm for 10 min. The aqueous phase was transferred to a new tube, 1 ml of 100% ice-cold ethanol was added to the tub and the samples were kept at − 20 °C overnight for DNA precipitation. The samples were then centrifuged at 13,000 rpm for 5 min, the supernatant was discarded and 1 ml of 70% ethanol was added to the tube. The mixture was centrifuged at 13,000 rpm for 2 min and the supernatant was discarded. The obtained pellet was oven-dried at 37 °C, and the DNA was resuspended in 50 μl of ultrapure water and eluted in a water bath for 30 min at 65 °C. The quantity and purity of each sample were estimated by spectrophotometry with a NanoDrop™ spectrophotometer (Thermo Fisher Scientific, Waltham, MA, USA), at absorbance readings of 260 nm and the 260/280 nm absorbance ratio, respectively. The 228 samples corresponding to each animal and collection time point were then stored at − 80 °C until further use.

### PCR analysis

These gDNA samples were analyzed by PCR in duplicate following previously described methodology [21] using the primers KB-18 (5′-GATGTACAACCTCACCAGAGTACC-3′ forward) and KB-19 (5′-CAACAAAATAGAACCAAGGTCCTAC-3′ reverse), which produce a PCR product of 262 bp. The PCR reaction was performed according to [10] using the following reagents: 2.5 µl of 10× buffer, 0.75 µl MgCl_2_ (50 mM), 0.5 µl dNTPs (2.5 mM; Invitrogen, Thermo Fisher Scientific, Waltham, MA, USA), 0.5 µl of forward and reverse primers (10 pmol), 0.3 µl *Taq* DNA polymerase (Ludwig Biotec, Rio Grande do Sul, Brazil), 1 µl of DNA (100 ng) and ultrapure water to a final volume of 25 µl. Two negative controls (a blood sample extracted from a healthy bovine donor and ultrapure water) and one positive control (a sample obtained from a bovine blood smear that tested positive for *B. bigemina*; Additional file 1: Image S1) were tested. The reactions were performed in a BioRad T100™ Thermal Cycler (Bio-Rad Laboratories, Hercules, CA, USA), and the cycling parameters were: 95 °C for 2 min, followed by 40 cycles of 95 °C for 1 min, 60 °C for 30 s and 72 °C for 1:30 min, with a final extension of 72 °C for 7 min.

The final product was visualized in a 1.5% agarose gel stained with ethidium bromide. Twelve samples yielding the expected PCR product size for *B. bigemina* were purified using a PureLink quick gel extraction kit (Invitrogen, Thermo Fisher Scientific). These DNA samples were sequenced at the Human Genome and Stem-Cell Research Center (Universidade de São Paulo [USP], São Paulo, Brazil) in an automatic sequencer (ABI 3730 DNA Analyzer; Applied Biosystems, Thermo Fisher Scientific, Waltham, MA, USA) with a 48-capillary DNA analysis system. The sequenced PCR products were analyzed with MEGA X software [22], and the consensus sequences of the analyzed samples were deposited in GenBank under accession number MZ542450.1.

### Real-time quantitative PCR analysis

Genomic DNA samples were diluted to a concentration of 100 ng/μl and subjected to real-time quantitative PCR (qPCR) analysis to quantify the circulating copies of the *B. bigemina *cytochrome* b* gene (*cbisg*) as described in [23]. Absolute quantification was performed using primers and double-quenched hydrolysis probes (PrimeTime® Std qPCR Assay; Integrated DNA Technologies, Coralville, IA, USA) based on the *B. bigemina* LK054939.1 sequence in GenBank and designed using the PrimerQuest Tool (Integrated DNA Technologies), which generated an 88-bp product of the *cbisg* gene (forward primer: 5′-TGTTCCAGGAGATGTTGATTCTT-3′; probe: 5′-/56-FAmQCGAGTGTGT/Zen/TATCAGAGTATTAACTGAGGT/3IABkFQ/-3′; reverse primer: 5’-GCACTTCGTTATTTCCATGCT -3′). Primer-dimer formation was tested with the OligoAnalyzer tool (https://www.idtdna.com/pages/tools/oligoanalyzer).

Specificity in silico was tested using the NCBI BLAST platform (https://blast.ncbi.nlm.nih.gov/Blast.cgi?PAGE_TYPE=BlastSearch). The organisms searched were limited to “bovine,” “human” and “babesia.”

The efficiency and reproducibility (Additional file 2: Table S1, Additional file 3: Figure S1, Additional file 4: Figure S2) of the reaction were calculated according to [24]. Serial dilutions (1:10) from 10^1^ to 10^10^ were used to construct a standard curve with different concentrations of synthetic DNA gBlocks® Gene Fragments (Integrated DNA Technologies) containing the sequence of *B. bigemina* (5′-TGACCTTTTATTATGTTCCAGGAGATGTTGATTCTTTCGAGTGTGTTATCAGAGTATTAACTGAGGTTAATATGGGTTGGGCACTTCGTTATTTCCATGCTCAATGTGTTTCTTTTTGCTTTTTCTTTATGATGTTACATATGTTAAAAGGTTTATG-3′—also constructed based on the sequence deposited as accession number LK054939.1). Positive controls and duplicate negative template and negative control samples were added to each qPCR run.

The reaction volume was 10 μl per well and consisted of 5 μl of Taqman™ Universal PCR Master Mix (Thermo Fisher Scientific), 0.5 μl of each primer (10 µM), 3 μl of Milli-Q H_2_O and 1 μl of 100 ng/μl gDNA. The reactions were run in duplicate. Ultrapure water was used instead of gDNA as a negative control.

A 5-point standard curve (concentrations of 10^5^ to 10^10^ gBlocks®) was used in triplicate as an internal control in each 98-well plate. The samples were analyzed using a StepOnePlus^™^ Real-Time PCR System (Thermo Fisher Scientific) using a hydrolase probe activation cycle of 95 °C for 10 min followed by 45 cycles of denaturation at 95 °C for 45 s and annealing/extension at 60 °C for 1 min.

The reaction signal was recorded during the extension step, and the data were analyzed using StepOne v2.3. The Minimum Information for Publication of Quantitative Real-Time PCR Experiments (MIQE) guidelines were followed [25].

Using the qPCR results, the number of target DNA molecules in each reaction (copy number [CN]) was calculated according to [26], as follows: CN (L) = (6.022 × 10^23^ [copies/mol] × concentration [g/mol])/molecular mass (g/l), where 6.022 × 10^23^ is Avogadro's number and the molecular mass is the average molecular weight of double-stranded DNA (330 × 2) multiplied by the size of the cloned fragment.

### Immunoassay

For antigen detection of anti-*B. bigemina* immunoglobulin G (IgG), the indirect ELISA (iELISA) technique was used following a protocol based on [27].

Total antigen from *B. bigemina* (produced by the Immunoparasitology Laboratory of the Faculty of Agricultural and Veterinary Sciences [FCAV]/São Paulo State University [UNESP], Jaboticabal, SP, Brazil) was diluted to an optimal concentration of 10 μg/ml in 0.5 M carbonate/bicarbonate buffer (pH 9.6). After 12 h of incubation at 4 °C, blocking was performed with phosphate buffered saline—Tween 20 (PBS—Tween 20) (pH 7.2, 0.05% PBST) and 6% powdered skim milk (Molico®; Nestlé, São Paulo, Brazil). The 96 well plates (Nunc Maxisorp™; Thermo Fisher Scientific) were incubated for 90 min at 37 °C in a moist chamber.

After three washes with PBST buffer, the positive, negative and reference sera were added (all diluted 1:400 in PBST + 5% normal rabbit serum). The plates were then incubated at 37 °C for 90 min in a moist chamber. After three washes with PBST, alkaline phosphatase-conjugated bovine anti-IgG (Sigma-Aldrich, St. Louis, MO, USA) diluted 1:30,000 in PBST + 5% normal rabbit serum was added, and the plates were washed again.

The alkaline phosphatase substrate p-nitrophenyl phosphate (Sigma-Aldrich) was then diluted in 1 mg/ml diethanolamine buffer (pH 9.8). The plates were sealed in aluminum foil and incubated for 30 min at room temperature, and then read at a wavelength of 405 nm on a micro-ELISA reader (B.T.-100; Embrabio, São Paulo, Brazil).

### Statistical analysis

R version 3.6.3 [28] and R Studio (8.15 build 180,091) [29] were used for statistical analysis.

The Kolmogorov–Smirnov test was performed to check data normality, and then the Mann–Whitney U-test was used to compare the weight, number of ticks and *B. bigemina cbisg* gene CN between the breeds because the data did not present a normal distribution.

The CNs and numbers of ticks were log_10_(n + 1)-transformed and then analyzed by Spearman’s rho statistic to estimate a rank-based measure of association among the weight, log_10_(CN) and log_10_(ticks). A *P*-value < 0.05 was considered to indicate statistical significance.

## Results

### Environmental data and bovine weighing

**Fig. 1:**
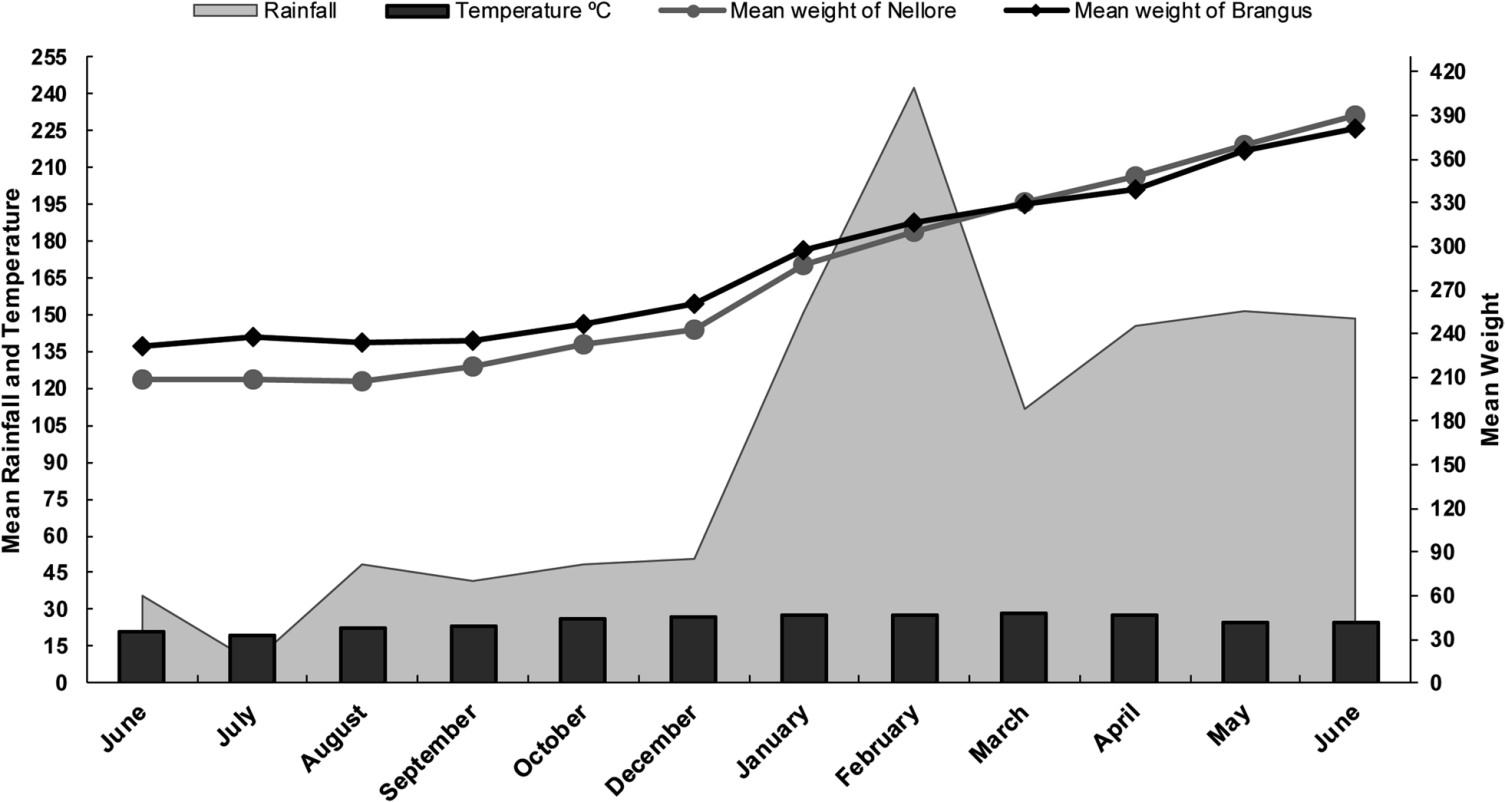
Mean rainfall (gray shading) and mean temperature (black bars) in the municipality of Água Clara, Mato Grosso do Sul (MS) State, Brazil. The gray line/gray circles indicate the mean weight (kg) of Nellore cattle, and the black line/black diamonds indicate the mean weight (kg) of Brangus cattle

During the sampling period, the mean environmental temperature was 24.95 ± 2.77 °C, the mean humidity was 66.86 ± 4.53% and mean rainfall was 33.41 ± 18.60 mm (Fig. 1).

The Brangus cattle had a mean (± standard deviation [SD]) monthly weight gain of 13.69 ± 8.1 kg, and the Nellore cattle had a mean weight gain of 16.46 ± 8.7 kg (Fig. 1). There was no significant difference between cattle types in mean weight at the start of the study (Brangus: 227.22 ± 17.26 kg; Nellore: 209.8 ± 24.36 kg) and at the end of the study (Brangus: 378.88 ± 31.59 kg; Nellore: 395 ± 40 kg) (Mann–Whitney U-test, *U*_(18)_ = 6126, *Z* = -0.698, *P* > 0.05).

### Tick count, PCR, qPCR and iELISA

**Fig. 2:**
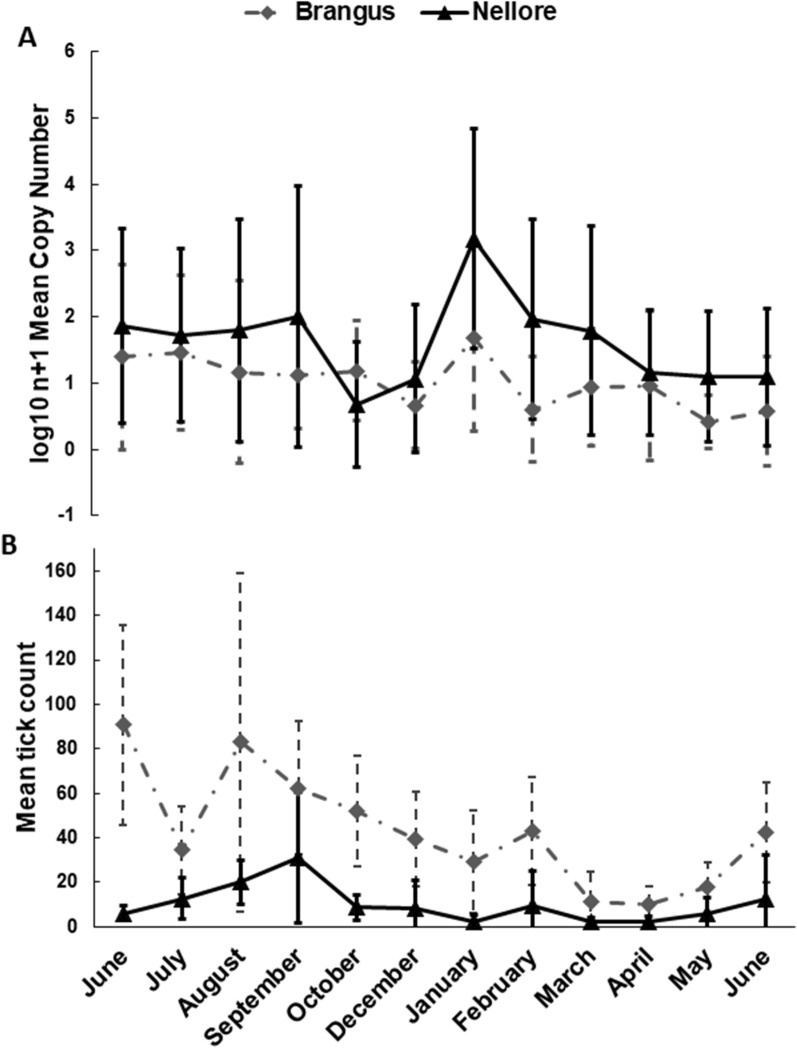
**A** Mean *Babesia bigemina* cytochrome* b* gene (*cbigs*) copy number (data subjected to log_10_ + 1 transformation) for Brangus (dashed gray line/gray diamonds) and Nellore (black line/black triangles) cattle.** B** Variation in mean *Rhipicephalus microplus* tick count for Brangus (dashed gray line/gray diamonds) and Nellore (black line/black triangles) cattle across 12 months

The mean (± SD) tick count was higher for Brangus cattle than for Nellore cattle (45.5 ± 20.9 vs 10.08 ± 2, respectively) (Mann–Whitney U-test, *U*_(18)_ = 2148, *Z* = -8.07, *P* < 0.01) (Fig. 2b). All samples tested by PCR and qPCR tested positive for *B. bigemina*; in contrast, but *B. bovis* was not detected in any of the samples tested by PCR and qPCR. Analysis of the circulating *B. bigemina cbisg* gene CN by qPCR (Fig. 2a) revealed a higher mean CN for the Nellore breed compared to the Brangus breed (3.25 ± 0.18 vs 2.5 ± 0.15, respectively) (Mann–Whitney U-test, *U*_(18)_ = 7775, *Z* = − 2.61, *P* = 0.005) (Table 1).

**Table 1:** Real-time quantitative PCR results for the detection of the *Babesia bigemina *cytochrome* b* gene

Parameters	E^a^	*R*^2^^2^	Slope	*Y*-intercept	PCR quantification number (Cq value)		DNA copies^c^	
					Nellore	Brangus	Nellore	Brangus
Maximum	103.18	1	3.349	41.89	38.10	37.27	4.91 ×10^5^	1.04 ×10^3^
Mean	99.0	0.990	3.345	38.14	32.72	34.10	2.23 ×10^2^	7.45 ×10^2^
Minimum	96.14	0.987	3.291	33.28	28.65	24.7	9.01 × 10^–1^	0 × 10^0^

^a^Efficiency of amplification

^b^Determination coefficient

^c^DNA copies expressed in log_10_ (*n* + 1)

Serological samples from each time point were analyzed by iELISA at a cutoff of 0.277. For Brangus cattle, 15/108 (13%) samples tested positive, and for Nellore cattle, 18/120 (15%) samples tested positive.

### Spearman correlation

For the Brangus breed, the Spearman's rank correlation coefficient (*rs*) = − 0.23 (*P* = 0.01) between weight and CN, − 0.47 (*P* = 0.00) between weight and tick count and 0.25 (*P* = 0.01) between tick count and CN. For the Nellore breed, Spearman's rank correlation coefficient (*rs*) = − 0.11 (*P* = 0.22) between weight and CN, − 0.42 (*P* = 0.00) between weight and tick count and 0.12 (*P* = 0.16) between tick count and CN.

## Discussion

Brazil is an endemic region for Bovine Parasite Sadness (BPS), and beef cattle breeders in the Cerrado biome have introduced other taurine breeds into the genetic lines of their herds to increase the production of beef cattle per hectare and to meet the demand from the consumer market for higher-quality meat [30]. However, this strategy has increased the sensitivity of the animals to ticks and increased the risk for BPS outbreaks.

The Água Clara region is characterized by three to four *R. microplus* generations per year, three of which occur in the rainy season from October to April, when most infestations occur [31]. Only a limited number of animals were examined in the present study due to the lack of use of acaricides and prophylactic control of TBDs. The tick counts were performed at 18-day intervals because the duration of the tick parasitic life-cycle is 21 days. Blood collections were made every 36 days because the life-cycle of *B. bigemina* has been estimated to be 4–5 weeks*.* Our data suggest that the high tick infestation rates in Brangus cattle in the initial month (June 2016) of the study may have been related to the stress generated at the end of the weaning period and the beginning of the growth phase combined with a lower pasture quality at this time of the year in the Cerrado biome [10] and subsequent nutritional stress, all of which suppressed the immune system of the cattle [32].

No significant difference in weight was detected between the two breeds during the experimental period, but a weak negative correlation was observed between the number of ticks and body weight. As reviewed by Jonsson [33], a negative effect on cattle weight caused by tick’s blood spoliation can be estimated for each engorged female as the loss of > 1 g in weight which, over time, would cause economic loss [34].

During the present study, larvae and nymphs were observed at the same infestation proportions in both breeds but were not quantified. Few engorged ticks were found on Nellore cattle, which led to the end of the parasitic phase. The resistance to ticks in cattle breeds like Nellore could be associated with increased numbers of mast cells, eosinophils and basophils in the skin, while the recruitment of neutrophils is potentially associated with tick susceptibility [6]. Increased numbers of mast cells, eosinophils and basophils cause the release of histamines from these cells, inhibiting tick attachment and leading to itching, increased grooming and tick removal [6].

Weak correlations between *Babesia* spp. CNs and tick counts have also been observed in a number of recent studies, with the results suggesting that there was no correlation between these factors at the time of data collection [35, 36]. However, Giglioti et al. [37] have suggested that a high positive correlation coefficient in bovine parasitemia may be dependent on or determined by the parasitemia burden for ticks.

Our results contradict previous data reported by Bilhassi et al. [38] on *Babesia* spp. which indicated that pure Zebu cattle should be expected to have a low number of ticks, resulting in relatively low levels of parasitemia. However, the methodology proposed by Wharton and Utech [17] is open to question as only the number of engorged parasites was taken into account, and nymphs also have the ability to transmit *Babesia* spp. [39] but are not counted in the methodology of Wharton and Utech [17].

*Babesia bigemina* can establish long-lasting chronic infections that are often accompanied by *Anaplasma marginale* and *B. bovis* infections, causing CTF. Even if a bovine host is able to establish an immune response that controls the disease, the parasite continues to proliferate in the bloodstream at levels that may be below detection by microscopy [40]. Although no clinical signs of babesiosis were observed during the present study, the possibility that subclinical cases were present cannot be ignored.

Água Clara is a region known to be endemic for CTF, and the farm on which the study was carried out is characterized by its use of an extensive production system with nursing, weaning and rearing phases.

Some studies have reported serological prevalence rates, such as 23% for *B. bigemina* in the State of Mato Grosso do Sul [41], 87.7–98.9% in the Pantanal region [42] and 97% in Pará State [43]. In the present study, we observed low iELISA responses (13% for Brangus cattle and 15% for Nellore cattle) that did not reflect the PCR and qPCR results. This result could be attributed to a state of equilibrium between *B. bigemina* and the host immune system (the cattle in this study were considered to be in good nutritional condition based on the weight gain curves) due to the ability to vary the antigens expressed on the surfaces of infected red cells, thus making the host a chronic carrier [44].

Enzootic stability of babesiosis in a herd occurs when the inoculation rate from ticks is sufficient to infect most calves before innate resistance to clinical disease disappears somewhere between 6 and 9 months of age, ensuring that most cattle are infected and immune before they reach an age at which they are susceptible to clinical disease [45]. However, Jonsson et al. [45] criticized the concept of enzootic stability and did not recommend its application to *Bos taurus indicus* or diseases that have inverse immunity because the experiments carried out by Mahoney and Ross [46] did not test these breeds and ticks, nor were serological assays performed. Thus, the degree of suppression of the host immune response in the field could not be evaluated as a function of infestation in the present study.

## Conclusions

In this study we analyzed field data from cattle in their growth phase raised in an extensive breeding system without acaricide treatment in an endemic environment, the Cerrado biome. The system might not have been the ideal system for these observations; however, the information gained contributes to an increased understanding of the seasonal dynamics of *B. bigemina* and may help lead to future identification and classification of strains that are less pathogenic to herds throughout this period. Although no correlation was detected between number of ticks and *B. bigemina cBisg* CN, the Nellore breed, even with a lower mean number of ticks, presented a higher mean CN than the Brangus breed. On the other hand, the two breeds showed similar weight development and no symptoms of babesiosis throughout the study period. More studies are needed to understand the dynamics between breed and the presence/quantity of *B. bigemina*. This was the first study performed on two cattle breeds in the rearing phase in the Cerrado biome with the aim to quantify tick counts and circulating *B. bigemina* CNs.

## Supplementary Information

**Additional file 1: Image S1.** Bovine blood smear stained with panoptic method observed through a 100× objective with immersion oil for *B. bigemina *detection.

**Additional file 2: Table S1.** Mean quantifying cycle and copy numbers obtained from each sample period for each breed.

**Additional file 3: Figure S1. **Quantification cycle (Cq) vs standard deviation (SD) for *Babesia bigemina* *cBisg* gene copies.

**Additional file 4: Figure S2.** Current cycle (CT) vs.* cBisg* double-strand gBlock quantity

## Data Availability

The majority of the data are included in the present manuscript. The nucleotide sequences are available under accession numbers LK054939.1 and MZ542450.1 in GenBank. All other relevant data are included in the manuscript and the references or are available upon request by the corresponding author.

## Abbreviations

CN: Copy number
CTF: Cattle tick fever
*cbisg*: *Babesia bigemina* cytochrome* b* gene
gDNA: Genomic DNA
iELISA: Indirect enzyme-linked immunosorbent assay
IgG: Immunoglobulin G
qPCR: Real-time quantitative PCR
TBD: Tick-borne diseases
