# Supplementary material for: Seasonal fluctuations of Babesia bigemina and Rhipicephalus microplus in Brangus and Nellore cattle reared in the Cerrado biome, Brazil
Source: Parasit Vectors. 2022 Oct 28;15:395. doi: 10.1186/s13071-022-05513-2 (PMC9617377; doi:10.1186/s13071-022-05513-2)
Supplement: Supplementary file 1 — Additional file 1: Image S1. Bovine blood smear stained with panoptic method observed through a 100× objective with immersion oil for B. bigemina detection. [file 13071_2022_5513_MOESM1_ESM.docx]

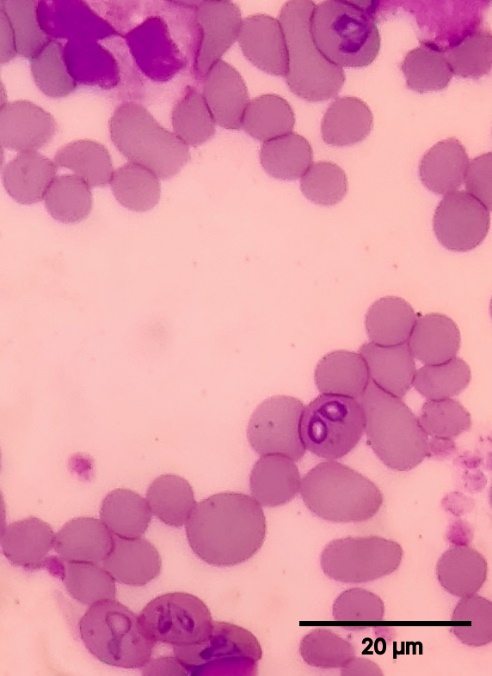


**Image S1:** Bovine blood smear stained with panoptic method observed in a 100x objective with immersion oil for *B. bigemina* detection.
