# Supplementary material for: Seasonal fluctuations of Babesia bigemina and Rhipicephalus microplus in Brangus and Nellore cattle reared in the Cerrado biome, Brazil
Source: Parasit Vectors. 2022 Oct 28;15:395. doi: 10.1186/s13071-022-05513-2 (PMC9617377; doi:10.1186/s13071-022-05513-2)
Supplement: Supplementary file 2 — Additional file 2: Table S1. Mean quantifying cycle and copy numbers obtained from each sample period for each breed. [file 13071_2022_5513_MOESM2_ESM.docx]

**Table S1:** Mean quantifiying cycle and copy number obtained from each sample period for each breed

|  | | **Nellore** | **Brangus** |
| --- | --- | --- | --- |
| **Sample month** | **Cq mean ± SE** | **Mean copy log ± SE** | **Mean copy log ± SE** |
| June | 30,30 ± 1,89 | 1,86 ± 0,46 | 1,39 ± 0,47 |
| July | 33,12 ± 0,69 | 1,72 ± 0,41 | 1,46 ± 0,39 |
| August | 34,03 ± 0,98 | 1,79 ± 0,53 | 1,16 ± 0,46 |
| September | 33,70 ± 1,00 | 2,0 ± 0,62 | 1,11 ± 0,26 |
| October | 34,68 ± 0,79 | 0,67 ± 0,30 | 1,18 ± 0,25 |
| December | 33,95 ± 0,70 | 1,06 ± 0,35 | 0,66 ± 0,22 |
| January | 31,80 ± 0,87 | 3,17 ± 0,52 | 1,67 ± 0,47 |
| February | 33,86 ± 0,75 | 1,96 ± 0,48 | 0,60 ± 0,27 |
| March | 33,59 ± 0,64 | 1,78 ± 0,50 | 0,93 ± 0,30 |
| April | 34,13 ± 0,73 | 1,16 ± 0,30 | 0,95 ± 0,38 |
| May | 34,55 ± 0,65 | 1,09 ± 0,31 | 0,41 ± 0,14 |
| June | 34,28 ± 0,67 | 1,09 ± 0,33 | 0,58 ± 0,27 |
