# Supplementary material for: Seasonal fluctuations of Babesia bigemina and Rhipicephalus microplus in Brangus and Nellore cattle reared in the Cerrado biome, Brazil
Source: Parasit Vectors. 2022 Oct 28;15:395. doi: 10.1186/s13071-022-05513-2 (PMC9617377; doi:10.1186/s13071-022-05513-2)
Supplement: Supplementary file 3 — Additional file 3: Figure S1. Quantification cycle (Cq) vs standard deviation (SD) for Babesia bigemina cBisg gene copies. [file 13071_2022_5513_MOESM3_ESM.docx]

**Figure S1:** Current cicle (CT) vs. cBisg double strand gBlock quantity
