# Supplementary material for: Seasonal fluctuations of Babesia bigemina and Rhipicephalus microplus in Brangus and Nellore cattle reared in the Cerrado biome, Brazil
Source: Parasit Vectors. 2022 Oct 28;15:395. doi: 10.1186/s13071-022-05513-2 (PMC9617377; doi:10.1186/s13071-022-05513-2)
Supplement: Supplementary file 4 — Additional file 4: Figure S2. Current cycle (CT) vs. cBisg double-strand gBlock quantity [file 13071_2022_5513_MOESM4_ESM.docx]

**Figure S2:** Quantification cycle (Cq) vs Standard deviation (SD) for *B. bigemina* *cBisg* gene copies.
